# Manipulation of Rat Movement via Nigrostriatal Stimulation Controlled by Human Visually Evoked Potentials

**DOI:** 10.1038/s41598-017-02521-6

**Published:** 2017-05-24

**Authors:** Bonkon Koo, Chin Su Koh, Hae-Yong Park, Hwan-Gon Lee, Jin Woo Chang, Seungjin Choi, Hyung-Cheul Shin

**Affiliations:** 1 School of Interdisciplinary Bioscience and Bioengineering, POSTECH, Pohang, Korea; 2 Department of Neurosurgery, Yonsei University College of Medicine, Seoul, South Korea; 3 Department of Physiology, College of Medicine, Hallym University, Chuncheon, Korea; 4 Department of Physical Education, Hallym University, Chuncheon, Korea; 5 Department of Computer Science and Engineering, POSTECH, Pohang, Korea

## Abstract

Here, we report that the development of a brain-to-brain interface (BBI) system that enables a human user to manipulate rat movement without any previous training. In our model, the remotely-guided rats (known as ratbots) successfully navigated a T-maze via contralateral turning behaviour induced by electrical stimulation of the nigrostriatal (NS) pathway by a brain- computer interface (BCI) based on the human controller’s steady-state visually evoked potentials (SSVEPs). The system allowed human participants to manipulate rat movement with an average success rate of 82.2% and at an average rat speed of approximately 1.9 m/min. The ratbots had no directional preference, showing average success rates of 81.1% and 83.3% for the left- and right-turning task, respectively. This is the first study to demonstrate the use of NS stimulation for developing a highly stable ratbot that does not require previous training, and is the first instance of a training-free BBI for rat navigation. The results of this study will facilitate the development of borderless communication between human and untrained animals, which could not only improve the understanding of animals in humans, but also allow untrained animals to more effectively provide humans with information obtained with their superior perception.

## Introduction

Brain-to-brain interfaces (BBI) link individual brains to achieve full-bandwidth communication^1^, without using formal language or gestures. In general, BBI requires cooperation between a brain-computer interface (BCI) and a computer-brain interface (CBI) such that the BCI is used to collect commanding brain signals from the sender and the CBI is used to transmit these signals to the target brain^1–4^. In addition to being useful for communication with other people, BBI could also be useful for communicating with animals, especially untrained ones^5^.

The first BBI was introduced by Vieira *et al*. in ref.^5^. This system transmitted the brain signals of an ‘encoder’ rat to match the cortical signals of the ‘decoder’ rat, which allowed the rats to share meaningful behavioural information. Subsequently, Yoo *et al*. showed the feasibility of using transcranial-focused ultrasound for BBI by inducing tail movement in anaesthetized rats based on mental commands from human^6^. Recently, Rao *et al.* and Grau *et al.* reported human-to-human connections using non-invasive BBI, respectively^7,8^, and Vieira *et al*. suggested the use of a biocomputer that uses multiple mouse brains^9^. Although there has been huge progress in BBI-based communication, all previous schemes between human and animal or humans required anaesthesia or training, respectively. In this reason, previous BBI systems have a difficulty to be used in practice for the communication between humans and untrained animals.

To tackle this limitation, we propose a novel BBI in which a person can manipulate an animal’s movement. A tremendous advantage of this system is that it does not require a training procedure. This new BBI is achieved by using a BCI that receives inputs from any person’s steady-state visually evoked potentials (SSVEPs) and a CBI that targets a rat’s nigrostriatal (NS) pathway. SSVEPs are electrical responses that occur in the brain when a person actively concentrates on a visual stimulus of a specific frequency. In general, SSVEP-BCIs recognizes a command by matching the user’s brain response and the flickering frequencies of the visual items^10,11^. This paradigm has been used for developing applications in speller, video games, and navigation^12–14^. The NS pathway is a part of the basal ganglia motor loop and is one of the brain’s primary dopaminergic pathways^15–17^. Rat models with a unilateral lesion of the NS have been shown to exhibit turning behaviour following injection of amphetamine or apomorphine, which is known to cause prolonged dopamine imbalance in the NS bundle by over-stimulating presynaptic terminals or by hyper-activating post-synaptic receptors, respectively^18,19^. This led us to hypothesize that electrical stimulation of the NS pathway via a CBI could be used to control locomotion in rats.

Here, we report a novel ratbot that can be controlled by electrical stimulation of the NS pathway directed by a human user. Our rat model exhibits an immediate contralateral turning behaviour in response to a single stimulation (e.g., stimulating the left-hemispheric NS induces a rightward turn). Thus, without any training, we can manipulate locomotion in our model rats by stimulating the pathway several times sequentially. Results showed a high success rate and speed, thus demonstrating the reliability of our proposed system. Moreover, our system showed no preference for direction. To our knowledge, this is the first reported use of NS stimulation in a ratbot model such as this in which ‘ratbot’ refer to a rat moves according to external commands^20,21^. Our empirical results imply that untrained human participants could make the untrained rats to successfully reach to the goal using the BBI system by means of their intention.

## Results

To construct our BBI system, we developed a novel ratbot model using NS pathway stimulation in addition to the controller with SSVEP-BCI. The controller enabled the human participants to selectively generate the SSVEP response implying the control command, and the system recognized the command by analyzing their brain activities. Then, the system transmitted a pattern of micro-electrical pulses to NS pathway of the rats through the stimulation electrodes implanted in their NS pathway; which induced the locomotion change in the rats as the human participants intended. Through this procedure, the BBI system allowed the communication between the humans and the untrained rats.

After the operation, each rat showed a unique response to identical stimulation. In the model validation procedure, average turning angles for rat 1, 2, and 3 were 98.0 ± 10.4°, 77.7 ± 28.5°, and 101.0 ± 34.4°, respectively to the left-hemispheric NS pathway stimulation, and 45.8 ± 8.4°, 61.7 ± 3.5°, and 73.7 ± 25.0°, respectively to the right-hemispheric NS pathway stimulation. We tried to minimize the difference in the behavioural response between ratbots by providing each ratbot with an individualized stimulation pattern. We thought that this was a better solution than simply modulating the stimulation amplitude, because an electrical stimulus with a high amplitude and/or a long duration can result in a dopamine level imbalance in the NS^22^. We did not quantitatively measure the effect of the patterns, but according to the report by Howe and Dombeck, different stimulation patterns can induce different locomotor behaviour^17^. In our opinion, the custom-designed stimulation patterns sufficiently influenced our empirical results.

Our target region is related to the midbrain dopamine neurons and is located close to the medial forebrain bundle (MFB). This suggests that electrical stimulation of the NS in our rat model could result in learning or addiction to the stimulation, as these are behaviours known to be related to activity in this circuitry. To clarify this issue, we performed an extra experiment in which the rats were trained in an operant conditioning task using a Skinner box^20,23^. In this experiment, our proposed rat model did not show any indications of addiction, nor did it learn to press the lever for intracranial self-stimulation. This suggested that our empirical results were not affected by the addiction and/or training effect of repetitive experiments, unlike the previous rat behavioural control model studies^20,23^.

**Figure 1:**
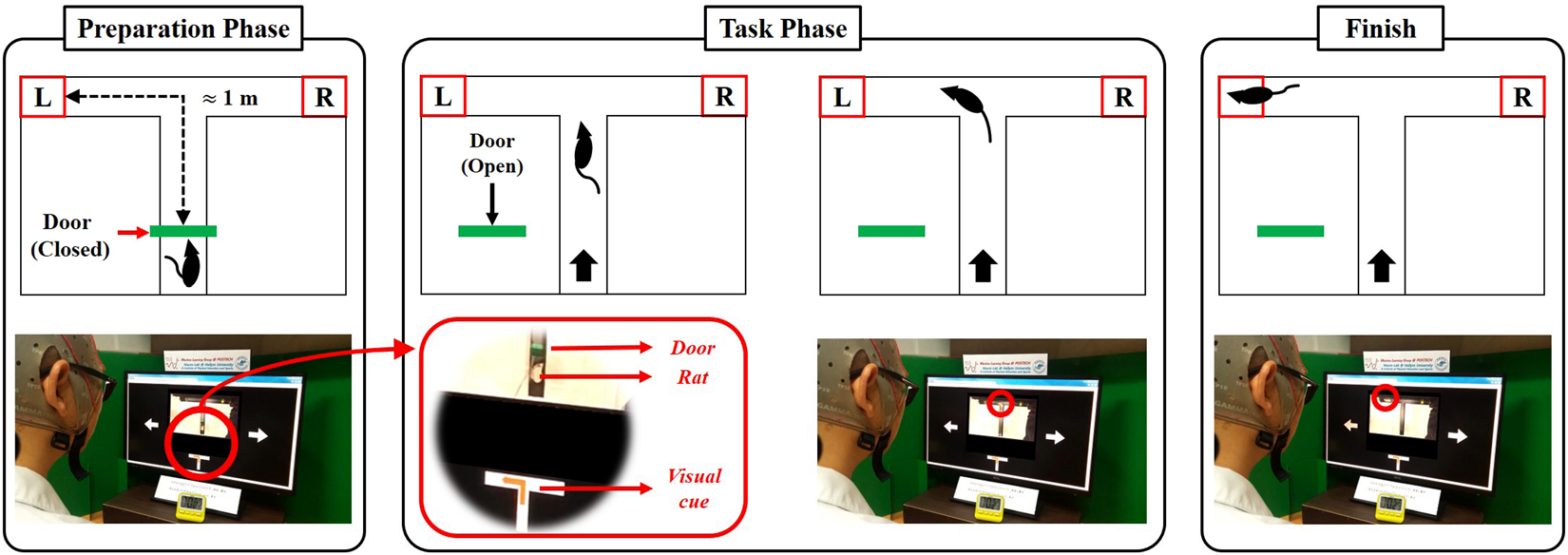
Schematic for the real-time rat-navigation experiment. Each experimental trial comprised a preparation phase and a task phase. In the preparation phase, experimenters set the system parameters, placed the rat at the start position, and checked the readiness of the human participant. The task phase began by flickering two directional arrows at different frequencies, displaying a live webcam image with a visual cue to the human participant, and opening the door of the maze. During the task phase, the human participant was required to move the rat to the goal, as determined by the visual cue. A trial ended when the rat arrived at one of the three ends of the maze (left, right, or the start position) after traversing the intersection.

To determine the success rate of the system, we conducted a real-time experiment that required participants to direct rats to the left or right end of a T-maze by commanding them with sequential brain signals. For this study, each participant controlled each rat 10 times (5 left turns and 5 right turns) over 1 or 2 days. The length of the experiment was decided by considering the fatigue of the participants (both human and rat). All experimental procedures were recorded in video clips, each of which documented five sequential experimental trials. Figure 1 displays a summary of the experimental task. We controlled a total of 180 samples from the six human participants and three rats. Each sample included the final destination of the rat for a trial. Success rates were calculated as the percentage of trials in which the rat reached the correct goal (Table 1). Overall, the average success rate was 82.2 ± 5.44%. Among the six participants, participant 4 had the highest success rate (90%; participant success range: 77–90%). The success rate for Rats 1 and 2 was 85%, and the Rat 3 was 76.7%. To assess the directional fairness of the proposed rat model, we measured and compared the success rates for left and right goals. Success rates for Rat 1, 2, and 3 were 80%, 80%, and 83.3%, respectively on the left task (L-task), and 90%, 90%, and 70.0%, respectively on the right task (R-task). The overall average success rate was much higher than random performance (33.3%), demonstrating the reliability of our proposed system. We compared the average success rates between two tasks and found that they differed by only 2.2%, which was less than the S.D. of either task. With this, we infer that our proposed model has no directional preference.

**Table 1:** Mean BBI success rates (%) for the T-maze task.

%	L-task				R-task				Overall
	Rat 1	Rat 2	Rat 3	Mean	Rat 1	Rat 2	Rat 3	Mean	
Hum 1	60.00	80.00	60.00	66.67	100.0	80.00	80.00	86.67	76.67
Hum 2	60.00	100.0	100.0	86.67	100.0	80.00	80.00	86.67	86.67
Hum 3	80.00	80.00	80.00	80.00	80.00	80.00	80.00	80.00	80.00
Hum 4	100.0	80.00	100.0	93.33	100.0	100.0	60.00	86.67	90.00
Hum 5	100.0	100.0	80.00	93.33	60.00	100.0	60.00	73.33	83.33
Hum 6	80.00	40.00	80.00	66.67	100.0	100.0	60.00	86.67	76.67
Mean	80.00	80.00	83.33	81.11	90.00	90.00	70.00	83.33	82.22
S.D.	17.89	21.91	15.06	12.23	16.73	10.95	10.95	5.58	5.44

**Figure 2:**
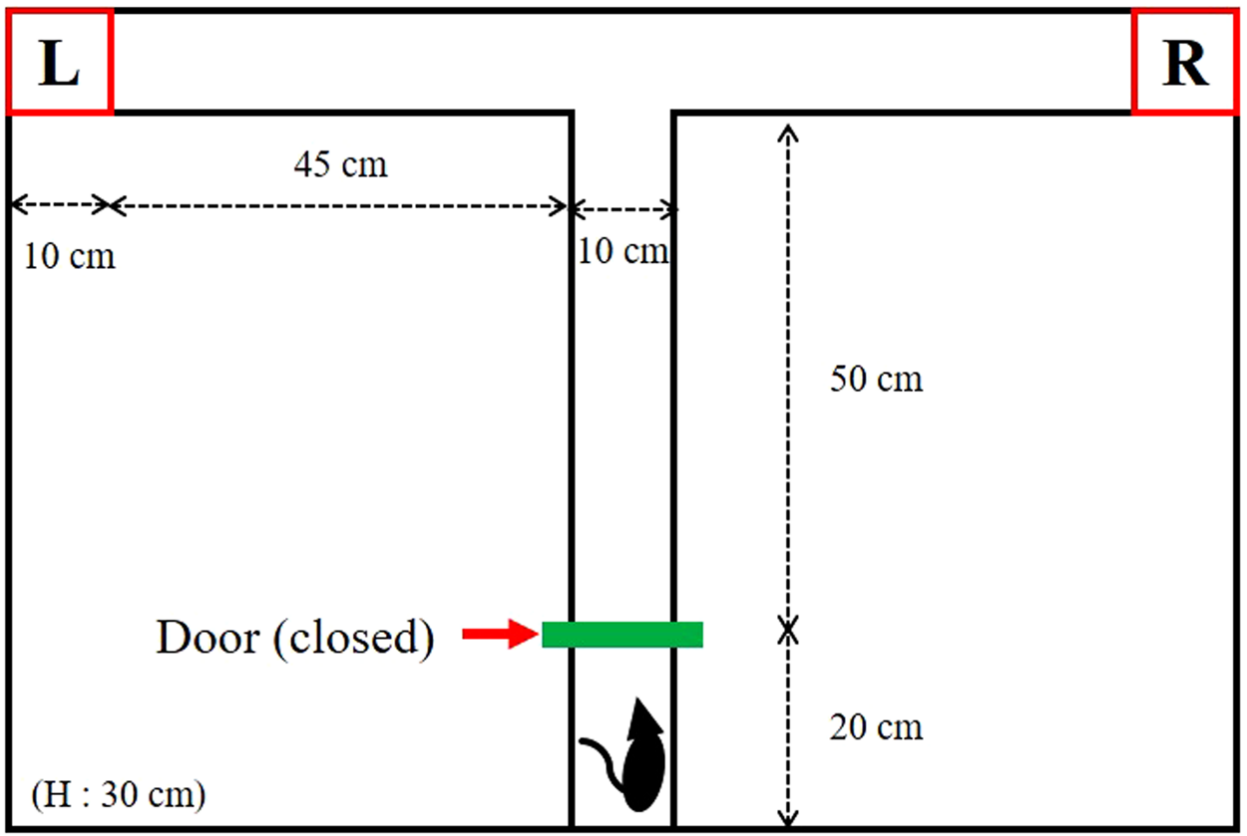
Design of the T-maze.

In this experiment, time to task completion is an important measure that indicates how much effort was required to the participants for completing a task; the system recognized a control command at every 0.5 s from the participants’ brain signals, so the number of commands sent by the participants is proportional to the time to complete the task. Table 2 lists the mean time to completion for each human-rat combination, as defined by the difference between the time when the rat reached the goal and the time when the researcher initiated the trial. Overall, the average time to completion was 30.3 ± 7.20 s. The average time needed for controlling Rats 1 and 3 was 17.1 s, while that for Rat 2 was 56.7 s. In particular, the combination of participant 6 and Rat 2 resulted in an exceptionally long times to completion (mean 86.4 s). Since the time to task completion is a variable which varies in the distance of the task, we have additionally calculated the speed of our ratbot model (also in Table 2), which is a more constant performance index to task change. To calculate this, we first estimated that the approximate distance the rat needed to move to complete the task was 1 m (based on the design of the maze, see Fig. 2). Then, we divided the distance by the overall average time needed to complete the task. As a result, the overall average speed was computed to be approximately 1.9 ± 0.49 m/min.

**Table 2:** Mean time for task completion (s) & speed of the rats (m/min).

	Time (s)				Speed (m/min)			
	Rat 1	Rat 2	Rat 3	Mean	Rat 1	Rat 2	Rat 3	Mean
Hum 1	18.2	68.2	18.1	34.8	3.2	0.8	3.3	1.7
Hum 2	19	63.5	14.4	32.3	3.1	0.9	4.1	1.8
Hum 3	16.9	34.4	24.1	25.1	3.5	1.7	2.4	2.3
Hum 4	16.4	49.5	16.3	27.4	3.6	1.2	3.6	2.1
Hum 5	17.6	37.9	7.7	21.1	3.4	1.5	7.7	2.8
Hum 6	14.3	86.4	22.2	41.0	4.1	0.6	2.7	1.4
Mean	17.1	56.7	17.1	30.3	3.5	1.0	3.5	1.9
S.D.	1.64	19.82	5.87	7.20	0.35	0.42	1.92	0.49

According to a report by Arbuthnott and Crow, an electrical stimulus can cause a brain damage in the NS pathway^22^. In this study, we tried to minimize the risk of brain damage resulting from the electrical stimulation. As a result, our ratbots showed stable life spans of at least one year. We developed our first ratbot in June, 2015 in an 8-week-old rat. As of Oct 2016, this ratbot is still alive with no disease symptoms, after going through more than 1 hour of experimentation per week. Our second and third ratbots also showed a stable life span, surviving ≥450 and 250 days after post-surgery, respectively.

## Discussion

In this study, we have demonstrated the successful development of a BBI between humans and untrained animals. Our system enabled human participants to manipulate rat locomotion with relative ease. Importantly, the system achieved this without needing to train the rats or the participants. To our knowledge, this is the first system to enable people to successfully communicate with untrained animals in this manner.

Our findings show that a training-free ratbot can be generated with electrical stimulation of the NS pathway. In 1971, Arbuthnott and Crow reported that this type of stimulation caused turning behaviour in rats^22^. From this report, we could formulate a hypothesis that sequential turning could change a rat’s position. Using this hypothesis, we developed a rat model in which we can forcibly control the rat’s locomotion.

In addition to these observations, we had a concern about the rat model when we first tried to develop the model. Because electrical stimulation cannot precisely specify target neurons, we were uncertain about the relationship between the behavioural response following electrical NS stimulation and the neural activity in the NS pathway. However, Howe and Dombeck recently reported that stimulation of dopamine axons in the substantia nigra can trigger locomotion in a mouse model^17^. With their report, we can confidently assume that the behavioural response is indeed induced by the NS stimulation, as the NS pathway connects the substantia nigra to the dorsal striatum^24^.

Interestingly, one of the three rats tested in this study required a longer time to complete the task under human control than the other rats. This was likely caused by rat-specific characteristics. Unlike the others, Rat 2 frequently exhibited voluntary movements that impeded completion of the task. For instance, it sometimes rotated in place or returned to the previous position after an induced movement. Therefore, the human controllers needed more time and effort, and ultimately sent more commands than were needed to control the other rats. Thus, although our empirical results demonstrate the reliability of our BBI system, this observation and others indicate several limitations. First, our system cannot control a voluntary behaviour of the rat during the inter-command interval. So, the rats could make unnecessary behaviours in the way of task, which resulted in delayed completion in some experimental trials. Second, when the participant’s command conflicted with the free will of the rat, some trials resulted in failure or longer control times. Lastly, our system performance did not improve over time as experimental trials increased (i.e., there was no learning curve)^23^. Although the human controllers reported that repetition facilitated their understanding of how to control the ratbots, this subjective effect was not obviously observed in our empirical results. This could have resulted from a limitation of the training-free nature of the system. In this study, the physiological mechanism of our rat model has not been deeply explored. Although the report by Howe and Dombeck has shown a relationship between locomotion and stimulation of dopaminergic axons in a mouse model^17^, to improve our ratbot model requires better understanding of the mechanism in rat. We thought that determining the mechanism will be an important research topic for a future study.

In conclusion, we report here a novel BBI system that enables humans to communicate with untrained animals using only brain activity. As the era of borderless communication is imminent, we believe that our model will provide a useful foundation for future BBI studies.

## Methods

### Participants

Six healthy humans were recruited for this study (mean age: 29 years). Participants 1, 2, and 3 had experience with an SSVEP-BCI, but the others did not. Before the experiment, we explained the experimental protocol to each participant and obtained written informed consent. All experimental procedures related to the human participants were conducted in accordance with the declaration of Helsinki. During the experiment, a comfortable chair was provided and participants were asked to maintain a constant posture to minimize motion-related noise. The experiment and procedures were approved by the institutional Review Board of Hallym University.

### Ratbot Model

**Figure 3:**
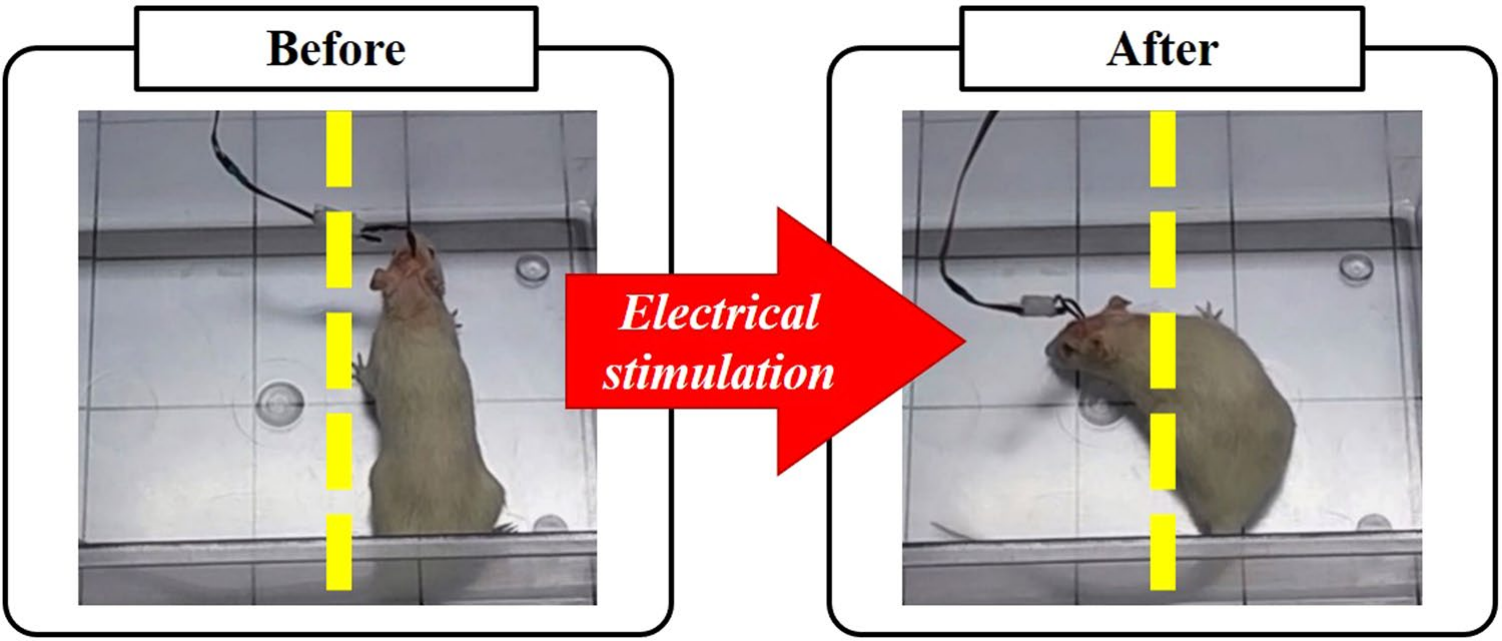
Change in the position of the ratbot in response to electrical stimulation of the right hemispheric NS pathway. The yellow dashed line is a reference for measuring the degree of turning and the amount of forward movement. With these images, we can see that electrical NS pathway stimulation can induce contralateral turning behaviour.

Three male rats (Sprague-Dawley, 300–400 g, Samtaco, Osan, Korea) were used for this study. All operational and experimental procedures related to the animals were approved by the Institutional Animal Care and Use Committee of Hallym University and performed in accordance with the Guide for the Care and Use of Laboratory Animals. We first anaesthetized the rats using a mixture of Zoletil 50 (40 mg/kg. Virbac S.A., France) and Rompun (10 mg/kg, Bayer), and the rat was placed on a stereotaxic apparatus (Stereotaxic Model 1404, David Kopf Instruments, USA). The rat was horizontally aligned using the bregma and lambda position and stimulation electrodes were implanted in the following manner. The bilateral coordinates of the NS pathway (AP, −3.8 mm; ML, ±1.6 mm; DV, 8.1 mm from the surface^25^) were confirmed on the skull and holes were drilled through the skull at their locations (Strong203, Saeshin Precision Co., Ltd., Korea); where the placement of the NS pathway was confirmed with the stimulation and histological evidence (see Supplementary Information file). Stimulation electrodes (customized pins; length, 12 mm; diameter, 300 *μ*m; polyimide coating, 0.008) and ground electrodes (screws; diameter, 340 *μ*m; precision screws & parts, Morris Co., USA) were then inserted into the target region using a manipulator. Finally, electrodes were fixed with dental cement, and an antibiotic (Tardomyocel Comp. III, 0.01 ml/100 g, Bayer AG Co., Germany) was applied to the surgical site. Implantation success was validated three days after surgery by stimulating the rat using an electric pulse generator (Model 2100, A-M systems. Calsborg, WA, USA). The pulse had a burst width of 200 ms, duration of 0.2 ms, period of 4 ms, and amplitude of 200 *μ*A. If a rat turned at least 30° following stimulation, we considered it to be a successful ratbot model. Figure 3 depicts a leftward behavioural response to electrical stimulation of the NS.

**Figure 4:**
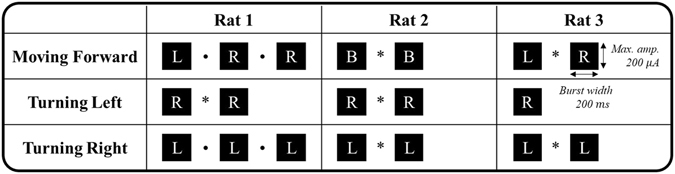
Individualized stimulation patterns for each command for each rat. Black squares represent a single electrical stimulation. The stimulation could be applied to the left (L), right (R), or both (B) NS pathways. The time between stimulations was either 200 ms (represented by a dot) or 300 ms (represented by an asterisk). All stimulation patterns were designed to be shorter than the inter-command interval of 500 ms. To compensate for response differences that depended on the rat’s condition, the stimulation amplitude (≤200 *μ*A) was selected before each experiment.

Because of individual variation between rats, optimal stimulation patterns for left and right turns and for forward movement were created through a trial and error process for each rat. We began with stimulation parameters set to a 50 *μ*A amplitude, 200 ms burst width, 0.2 ms duration, and 4 ms period. Then, we sequentially stimulated left, right, or both NS pathways one to three times and monitored the behavioural responses. If the rat turned at least 15° for a turning command or moved forward at least one step for a forward command, the response was considered significant and we saved the parameters excepting the amplitude as an individualized stimulation pattern. Otherwise, we increased the stimulation amplitude by 5 *μ*A and repeated the process. Figure 4 gives the individualized stimulation patterns for each rat-command pair.

### Configuration of the BBI

**Figure 5:**
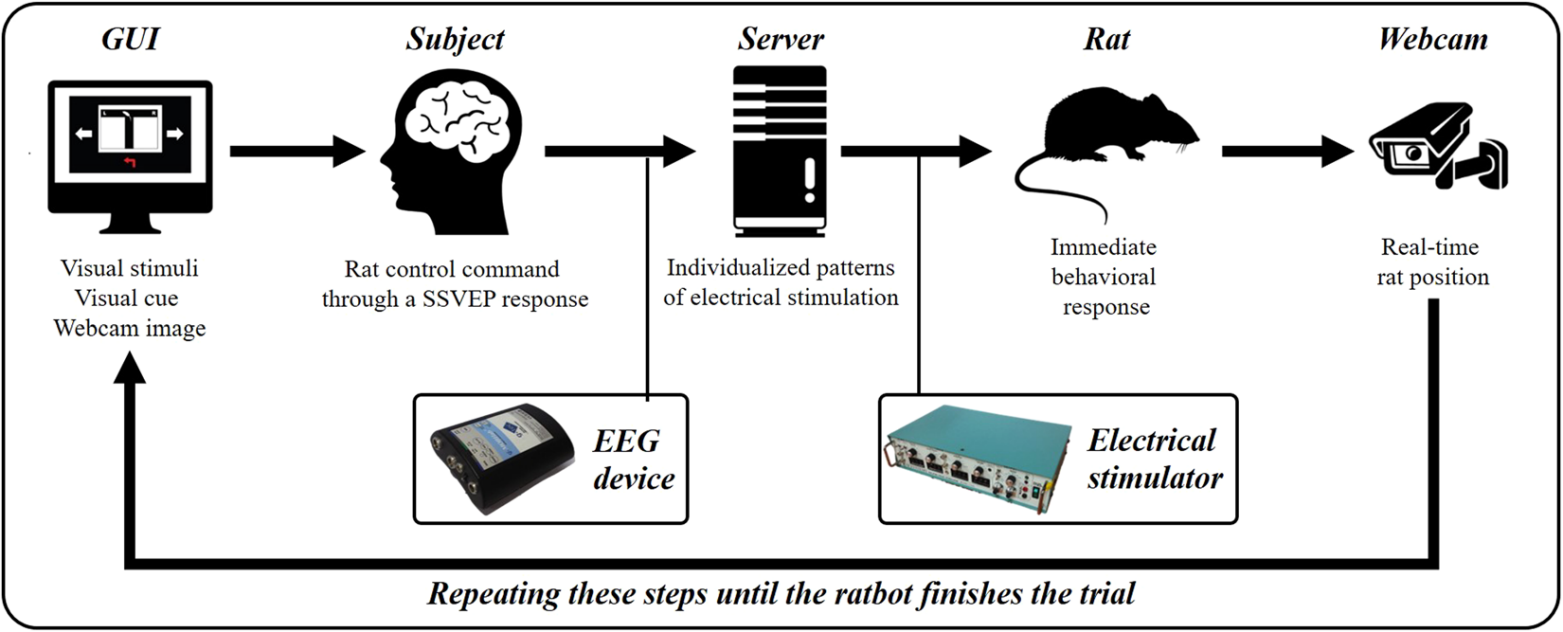
Schematic overview of the proposed rat-navigation system. This overview explains how information flowed between the human participant and the ratbot during an experimental trial. Two directional arrows on the GUI flickered at different frequencies to induce the SSVEP response from the participant. At the same time, the visual cue on the GUI indicated the goal for the current trial. To make the ratbot move in the intended direction, the participant selectively generated the SSVEP response by concentrating on one of the flickering arrows or ignoring both. If three sequential SSVEP responses corresponded to the left or right flickering arrow, they were considered to be a command to turn left or right, respectively. If not, they were considered to be a command to move forward. The server recognized the command by analysing the EEG signals, and then delivered the appropriate customized stimulation pattern through the neural stimulator to the electrodes implanted in the rat’s NS pathways. Because the maze was displayed in real-time on the computer monitor, the participant could recognize when and how the patterned stimulation induced the behavioural response from the rat. These steps were repeated until the experimental trial has been completed.

The BBI system comprised an electroencephalography (EEG) device, a server, a brain stimulator, and a graphical user interface (GUI). The EEG device monitored the participant’s brain activity, while the GUI provided the participant with visual stimuli. The server recognized the participant’s commands by analysing the recorded EEG signals. The brain stimulator provided the rats’ NS pathways with the patterned electrical stimulation according to the recognized command. The rat movement induced by this process was displayed on the GUI for the participant to see. Figure 5 shows a flow chart for our BBI system.

### Brain Imaging Device

An eight-channel EEG (g.MOBIlab, g.tec, Austria) device was used in this study. The device sampling rate was set to 256 Hz. The electrodes were placed on the participants’ scalps above the visual cortex according to the international 10–20 system (Cz, CPz, Pz, POz, PO3, PO4, PO7, and PO8). Reference and ground electrodes were located at the forehead and left earlobe, respectively.

### Graphic User Interface

**Figure 6:**
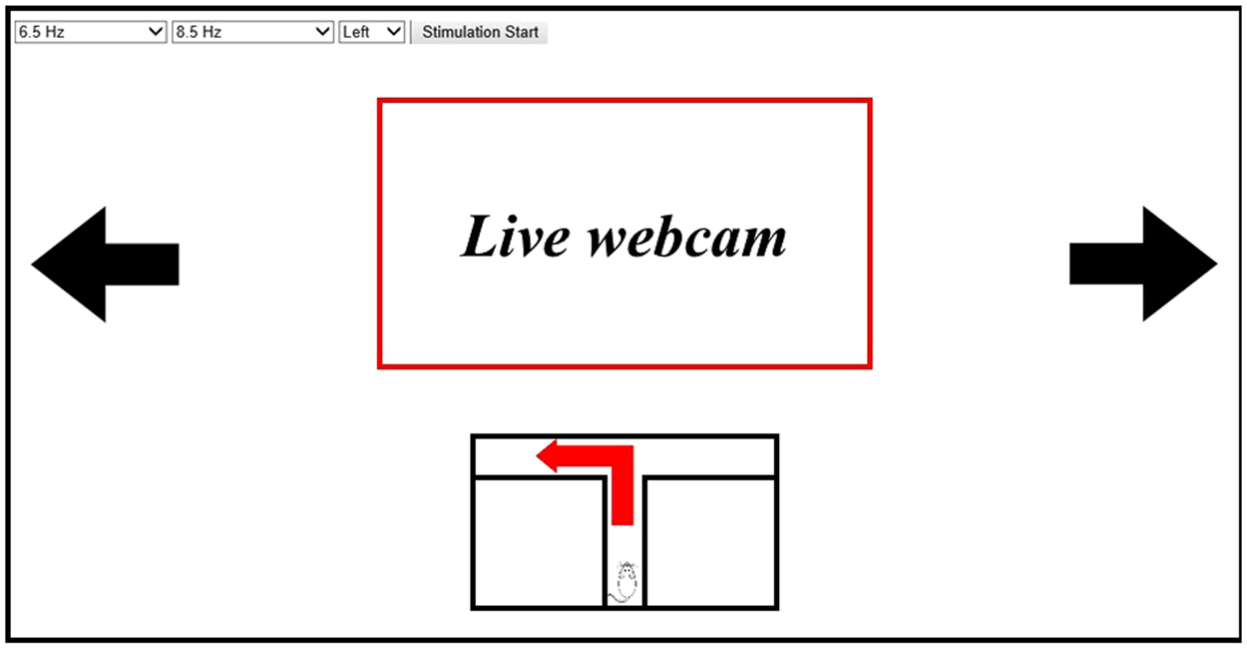
Design of the GUI.

We designed a GUI with a black background (Fig. 6). A control panel was located at the top-left side of the GUI. The control panel comprised three pop-up menus and a start button. Two pop-up menus were for selecting the stimulation frequencies, and the other was for selecting the task. After setting all parameters, the start button was activated. The experimental manager started the trial by clicking the start button. A leftward arrow was located on the left side of the GUI. During the experimental trial, it acted as the visual stimulus needed to generate the SSVEP signal by flickering at the frequency selected with the first pop-up menu. A rightward arrow functioned similarly. A webcam viewer was located in the centre of the GUI. During the experimental trial, streaming video showed the rat inside of the maze in real-time. A schematic of the maze was located at the bottom of the GUI. Before the experimental trial, it showed a rat at the start position. When a trial began, a leftward or rightward red arrow appeared inside the schematic to indicate the goal for that trial.

### SSVEP Detection Method

Canonical correlation analysis (CCA) is a method that estimates the underlying correlation between two sets of time-series data^26^. Given the two sets of time-series EEG signals (*X*) and the sinusoidal reference of f Hz (*Y*_*f*_), CCA finds the linear weight vectors (*w*_*x*_ and *w*_*y*_) that maximize their underlying correlation (*ρ*_*f*_); where the underlying correlation can be described by the following equation:

$${\rho }_{f}=\frac{E[{w}_{x}^{T}{X}^{T}{Y}_{f}{w}_{y}]}{\sqrt{E[{w}_{x}^{T}{X}^{T}X{w}_{x}]E[{w}_{f}^{T}{y}_{f}^{T}{Y}_{f}{w}_{y}]}}.$$

Because *ρ*_*f*_ approximates the power of the *f* Hz SSVEP response in *X* by comparing the *ρ*s obtained with the varying frequencies, we can estimate the frequency of the SSVEP response that exists in *X*. This method has been widely used in BCI studies for detecting the SSVEP response from EEG signals because of this phenomenon^27^. Our system detected the SSVEP response from the last 1.5 s of the EEG signal every 0.5 s. Because our system used two different stimulation frequencies (6.5 Hz and 8.5 Hz, one for each arrow) two different underlying correlations (*ρ*_6.5Hz_ and *ρ*_8.5Hz_) were estimated from the EEG segment for each detection. Therefore, we compared the correlations and accepted the SSVEP response corresponding to the higher underlying correlation. Each classification result was then stored in the system, and the last three classification results were recognized as the command signal. For instance, when the last three results all showed an SSVEP response of 6.5 Hz corresponding to the left arrow, the system recognized the sequence of results as the command to turn left. Therefore, the subject needed approximately 2.5 s (= 1.5 + 0.5 × 2) to turn the rat left or right. If the last three results were not identical, the system recognized the sequence of results as the command to move forward. Thus, the participant could move the rat forward by inhibiting the SSVEP response corresponding to the arrows, i.e., concentrating to the streaming video. After the commands were recognized, the system relayed them to the rat by electrically stimulating its NS pathway with the appropriate individualized stimulation pattern via the brain stimulator.

## Electronic supplementary material

Supplementary information file

Supplementary Video 1. The rat navigating experiment

Supplementary Video 2. The open maze test

Supplementary Video 3. The turning behaviour test
