# Publisher Correction: Manipulation of Rat Movement via Nigrostriatal Stimulation Controlled by Human Visually Evoked Potentials

**DOI:** 10.1038/s41598-017-14994-6

**Published:** 2017-12-19

**Authors:** Bonkon Koo, Chin Su Koh, Hae-Yong Park, Hwan-Gon Lee, Jin Woo Chang, Seungjin Choi, Hyung-Cheul Shin

**Affiliations:** 1 School of Interdisciplinary Bioscience and Bioengineering, POSTECH, Pohang, Korea; 2 Department of Neurosurgery, Yonsei University College of Medicine, Seoul, South Korea; 3 Department of Physiology, College of Medicine, Hallym University, Chuncheon, Korea; 4 Department of Physical Education, Hallym University, Chuncheon, Korea; 5 Department of Computer Science and Engineering, POSTECH, Pohang, Korea

*Scientific Reports* **7**:2340; doi:10.1038/s41598-017-02521-6; Article published online 24 May 2017.

In the original version of this Article, Jin Woo Chang was incorrectly listed as being affiliated with ‘School of Interdisciplinary Bioscience and Bioengineering, POSTECH, Pohang, Korea’.

The correct affiliation is listed below:

Department of Neurosurgery, Yonsei University College of Medicine, Seoul, South Korea.

This error has now been corrected in the HTML and PDF versions of the Article.

