# Supplementary material for: Manipulation of Rat Movement via Nigrostriatal Stimulation Controlled by Human Visually Evoked Potentials
Source: Sci Rep. 2017 May 24;7:2340. doi: 10.1038/s41598-017-02521-6 (PMC5443769; doi:10.1038/s41598-017-02521-6)
Supplement: Supplementary file 1 — Supplementary information file [file 41598_2017_2521_MOESM1_ESM.pdf]

Supplementary Information

Manuscript Title: Manipulation of Rat Movement via Nigrostriatal Stimulation Controlled by Human Visually Evoked Potentials

Author List: Bonkon Koo, Chin Su Koh, Hae-Yong Park, Hwan-Gon Lee, Jin Woo Chang, Seungjin Choi and Hyung-Cheul Shin

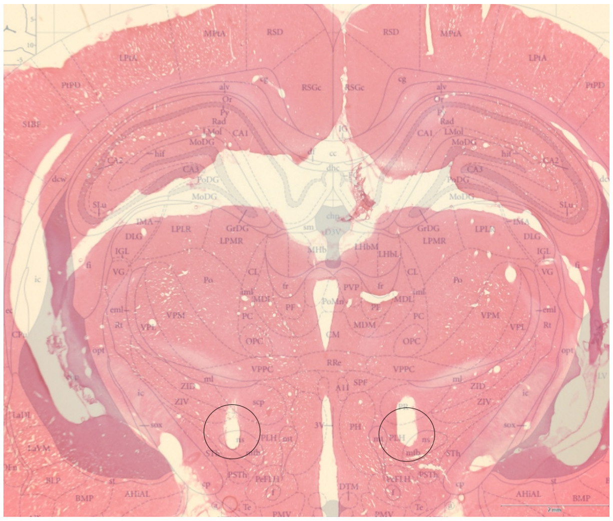

Supplementary Figure 1. Histology and H&E staining image. The surface image of the rat brain processed by using hematoxylin and eosin (H&E) staining displayed on the background of the Rat Brain Atlas. Two black circles indicate that the tip of each stimulation electrode was located in the NS pathway of each hemisphere.

Supplemenatry Video 1. The rat navigating experiment. The human participant manipulates the locomotion in the rat at three experimental trials according to sequential visual cues by means of commanding brain signals. Where the visual stimuli provided at the sides of the GUI enable the participant to generate the commanding brain signals selectively.

Supplemenatry Video 2. The open maze test. The human experimental manager, who does not appear on the scene, controls the rat to walk across the center of the open maze, which is the brightest spot of the maze, by using the two-button controller.

Supplemenatry Video 3. The turning behaviour test. The human experimental manager, who does not appear on the scene, repeatedly stimulates the NS of the rat using the two-button controller, and the rat responds to each stimulation by showing a contralateral turning behaviour.
